# The effectiveness of outbreak response measures for pathogens of different characteristics

**DOI:** 10.1038/s41598-026-64138-y

**Published:** 2026-08-10

**Authors:** Fenella McAndrew, Romesh G Abeysuriya, Nick Scott

**Affiliations:** 1 Burnet Institute, Melbourne, Australia; 2 Department of Epidemiology and Prevention Medicine, Monash University, Melbourne, VIC Australia

**Keywords:** Agent-based model_1_, Outbreak_2_, Pathogens_3_, Disease characteristics_4_, Non-pharmaceutical interventions_5_, Computational biology and bioinformatics, Diseases, Health care, Mathematics and computing, Microbiology

## Abstract

The initial response to an outbreak of a novel pathogen requires implementing measures to limit transmission before its characteristics and behaviour are fully understood. This study aimed to investigate how the effectiveness of key public health interventions may depend on pathogen characteristics. Using an agent-based model we simulated outbreaks of pathogens with different R_0_, latent period, infectious period and symptomatic fraction, under different public health policies (e.g. public health and social measures, testing, tracing, isolation and quarantine duration and mask usage). For each scenario and pathogen, we recorded infections over the first 60 days and in total, and calculated relative reductions compared to a no-intervention baseline. We found that R_0_ was the primary driver of total outbreak size. The latent period influenced the outbreak speed, and hence how quickly and for how long a public health response may be needed. A longer infectious period and higher symptomatic fraction increased the relative impacts of testing, tracing, isolation and quarantine. Compared to testing and tracing scenarios, public health and social measures were less sensitive to symptomatic fraction and infectious period. These results demonstrate the importance of rapidly identifying these disease characteristics to guide more targeted and efficient policy responses.

**Supplementary Information:**

The online version contains supplementary material available at https://doi.org/10.1038/s41598-026-64138-y.

## Introduction

New emerging pathogens with pandemic potential are a great concern for public health. Previous pandemics have claimed millions of lives and devastated economies. Over the last century there have been many major pandemics, most contributing to millions of deaths and lasting for multiple years^1^. Pandemics are likely to continue to occur with increasing frequency, driven by global travel, urbanisation, climate change and increased human-animal contact^2^.

When a new pathogen emerges, very little is known about the disease, but governments and public health organisations are required to make decisions on how to respond. The early days of a pathogen are critical, as the effectiveness of responses could dictate whether an infectious disease outbreak becomes a pandemic^3^. The basic reproduction number (R_0_) has been identified as one of the most important early characteristics to know about a disease, as it defines the early spread particularly within households^4,5^. However, other pathogen characteristics such as latent period, infectious period and symptomatic fraction could be critical to learn in the early stages as they may have the potential to impact the effectiveness of interventions. Due to the potential economic burden, as well as impacts to people’s mental health and wellbeing, it is necessary that interventions that are chosen are the most efficient for a given pathogen to avoid unnecessarily harsh restrictions^6^.

At the start of the COVID-19 pandemic, without any vaccines or antivirals available, governments rapidly implemented a range of non-pharmaceutical interventions, beginning with border closures, travel restrictions and quarantine measures for people entering different countries^7^. As the pandemic continued, mask-mandates, mass testing, contact tracing and banning of mass gatherings were introduced^8^. Quarantine was one of the first interventions introduced during pandemic, with many studies conducted investigating the efficacy for preventing COVID-19 spread^9–11^. An early recommendation was to disinfect surfaces as it was assumed early on that COVID-19 was highly transmissible via surfaces^12^. However, once it was established that COVID-19 was an airborne pathogen this recommendation was withdrawn, and masks were identified as an effective measure for decreasing onwards transmission^13,14^. Other public health measures such as school closures and lockdowns were some of the most effective at reducing transmission^15,16^, however they had a massive negative impact on the economy and individuals’ wellbeing^6,17^. Lessons learned from COVID-19 will be integral in informing early responses to future novel pathogens.

There is detailed work that has been done on how different pathogen characteristics may impact disease spread, particularly with compartmental models^18^. However, there is a gap in the literature in understanding the interaction between interventions and disease characteristics. While certain interventions proved effective for controlling the spread of COVID-19, it is not guaranteed they will be as effective in the early stages of a different pathogen. Currently it is unknown how effective different strategies would be for different pathogen types, and what strategies are best to contain outbreaks of novel pathogens in the early stages. Research methods will need to be developed to understand the interplay between interventions and disease characteristics.

Mathematical modelling is a valuable tool for investigating the spread of infectious diseases and the effectiveness of interventions to control them. Compartmental models are among the most common when modelling pathogen spread in a population^19,20^, with the Susceptible-Infected-Recovered (SIR) and Susceptible-Exposed-Infected-Recovered (SEIR) frameworks frequently used to model airborne pathogens (such as COVID-19, Influenza, measles)^21–23^. These models have been used to understand the impact of different disease characteristics such as R_0_, latent period and infectious period on outbreak outcomes, but are limited in their ability to capture network-specific interventions such as contact tracing^18,24,25^. Mathematical models have proved paramount in setting up countries for pandemic preparedness^26,27^.

By expanding on previous research using compartmental models, and instead using agent-based modelling techniques, it is possible to more accurately model interventions such as contact tracing that are likely to be highly utilised when responding rapidly to future pathogens. Furthermore, while previous studies have typically analysed the outbreak curve over the entire course of an epidemic, it is likely that bespoke models will be developed as more is learned about the pathogen. However, when a novel pathogen first emerges, such tailored models will not be available. In these early stages, pre-existing generalised models designed to guide initial responses will be highly valuable.

The aim of this work is to estimate the effectiveness of different policy options (testing, contact tracing, quarantine, isolation and different public health and social measures) for controlling outbreaks of pathogens of different characteristics using agent-based modelling techniques. This analysis is focused on airborne pathogens that are epidemiologically similar to SARS-CoV-2. This approach allows us to reuse context-specific data from the COVID-19 pandemic, and reduce the scope of possible pathogens considered.

## Methodology

### Model overview

An established agent-based model, Starsim^28^, was used to simulate outbreaks of novel pathogens with different characteristics. A synthetic population of 50,000 agents was used as simulations with 100,000 agents had the same modelled outcomes but with much greater runtime. The model has already been used to simulate outbreaks of Ebola, measles, cholera, yellow fever, and meningococcal meningitis^29^.

**Fig. 1:**
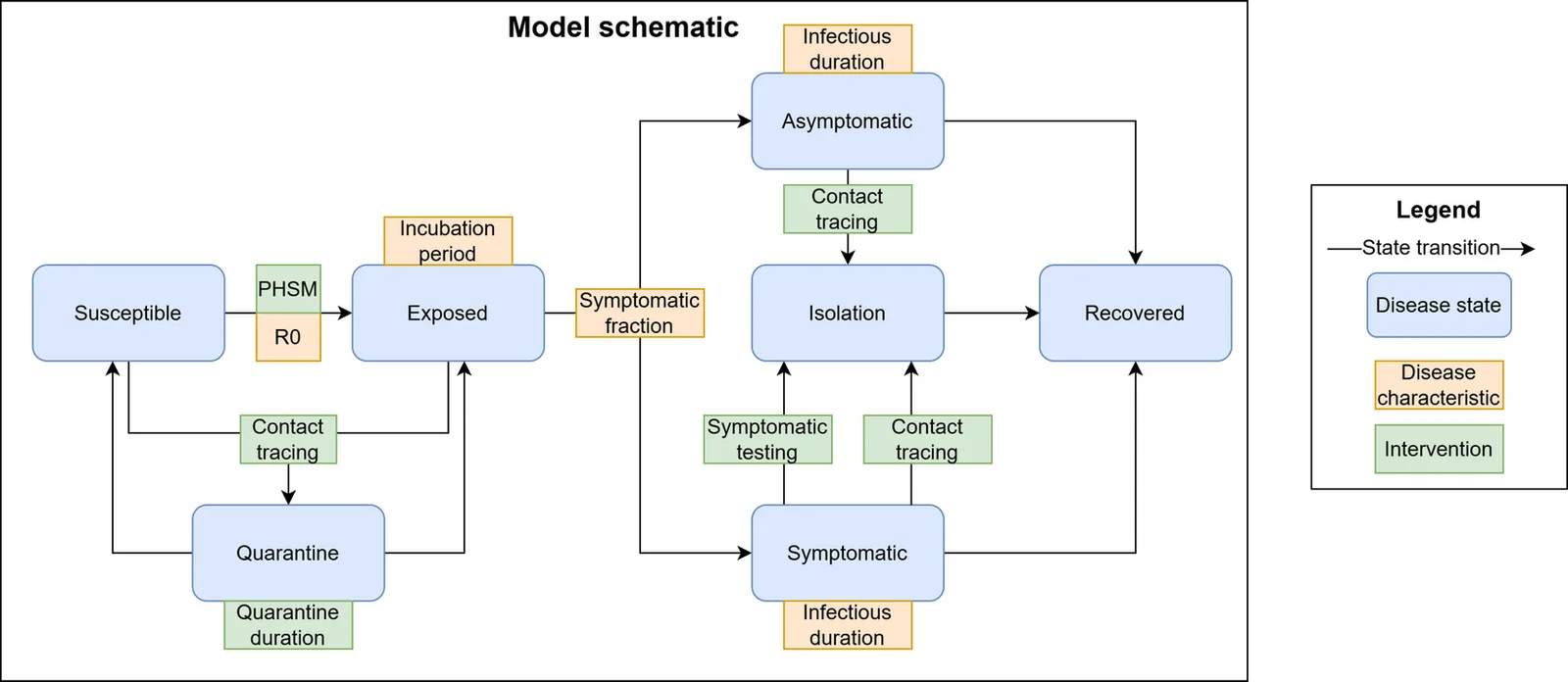
SEIR model schematic with disease characteristics and interventions.

Agents in the model were classified according to disease state (susceptible, exposed, infectious-symptomatic, infectious-asymptomatic or recovered), quarantine status (yes/no) and isolation status (yes/no) (see Fig. 1). For this study we assumed infectious-symptomatic and infectious-asymptomatic individuals had the same relative transmissibility.

Hospitalisation and deaths from the new pathogen were not considered, as the analysis considers the early stages of an emergent pathogen on the basis that it is of great enough concern to try and control the spread by considering the relative effectiveness of policies.

For this analysis we only considered airborne pathogens as interventions and their corresponding effectiveness were taken from previous COVID-19 studies (Table 1).

**Table 1:** Modelling input parameters, including disease characteristics, intervention assumptions and network specific parameters.

Parameter area	Estimate	Source
Disease characteristics		
Symptomatic fraction range	0 to 1	Total possible range
Incubation period range	2 to 14 days	Likely range [35]
Infectious period range	2 to 14 days	Likely range
R_0_ range	1 to 18	Covers range of possible diseases
Networks structure		
Household		
Mean household size	3	Scott et al., Supplementary material [37] Sampled as a normal distribution
Social		
Relative transmission risk compared to household contacts	0.12	Scott et al., Supplementary material [37]
Network applies to	All	Model assumption
Mean contacts	6	Scott et al., Supplementary material [37] Sampled as a Poisson distribution
Work		
Relative transmission risk compared to household contacts	0.28	Scott et al., Supplementary material [37]
Network applies to	18- to 64-year-olds	Working age
Mean contacts	5	Scott et al., Supplementary material [37] Sampled as a Poisson distribution
Proportion working aged employed	61%	Australian Bureau of Statistics employment data [38]
Schools		
Relative transmission risk compared to household contacts		
Classroom	0.25	Scott et al., Supplementary material [37]
Teacher – teacher	0.25	Scott et al., Supplementary material [37]
Student – student	0.028	Scott et al., Supplementary material [37]
Size		
Mean school size	298	Scott et al., Supplementary material [37] Sampled as a normal distribution
Mean class size	22	Scott et al., Supplementary material [37] Sampled as a normal distribution
Mean non-classroom staff-staff contacts	5	Scott et al., Supplementary material [37] Sampled as a Poisson distribution
Mean non-classroom student-student contacts	2	Scott et al., Supplementary material [37] Sampled as a Poisson distribution
Testing interventions		
Symptomatic testing		
Probability of having a test	75%	Optimise report [39]
Delay from symptom onset to testing	2-days	Office for National Statistics, UK [40]
Test sensitivity	100%	Model assumption
Test specificity	100%	Model assumption
Contact tracing		
Time between test being taken and commencement of contact tracing	1 day	Model assumption
Probability that a contact within this network can be traced		
Household	0.9	Scott et al., Supplementary material [37]
Social	0.5	Scott et al., Supplementary material [37]
Work	0.5	Scott et al., Supplementary material [37]
School	0.9	Scott et al., Supplementary material [37]
Isolation		
Isolation compliance	1	Model assumption
Isolation duration	Until agent is no longer infectious	Model assumption
Quarantine		
Quarantine compliance	1	Model assumption
Social measures		
Mask wearing in isolation of other interventions		
Relative reduction in transmission through social contacts	30%	Scott et al., Supplementary material [37]
Relative reduction in transmission through work contacts	30%	Scott et al., Supplementary material [37]
Relative reduction in transmission through school contacts	30%	Scott et al., Supplementary material [37]
Work from home		
Workplace attendance reduction	45%	Scott et al., Supplementary material [37]
Household transmission increase	10%	Scott et al., Supplementary material [37]
School closure		
Ages stay at home	12+	Scott et al., Supplementary material [37]
Student attendance	9%	Scott et al., Supplementary material [37]
Teacher attendance	9%	Scott et al., Supplementary material [37]
Mask wearing ages for students still attending school	8+	Scott et al., Supplementary material [37]
Mask transmission reduction	30%	Scott et al., Supplementary material [37]
Lockdown		
Work from home	Yes	Scott et al., Supplementary material [37]
School closure	Yes	Scott et al., Supplementary material [37]
Social transmission reduction	40%	Scott et al., Supplementary material [37]

### Demographics and contact networks

The age and household size distribution is drawn from Australian data from the state of Victoria used in previous work (see Table 1), and is broadly representative of high-income urban settings.

The model includes four contact networks: household, school, work and social networks. The work network is implemented as a collection of fully-connected workplace clusters, with working-age individuals assigned to workplaces based on the proportion of the 18-64-year-old population that are employed. Schools are implemented as collections of fully-connected classroom clusters with a teacher assigned to each classroom, and additional random mixing between teachers and students in different classes within the same school. The social network is a random network and is used to represent both social and random daily contacts.

The focus of this study is on the interaction between pathogen characteristics and intervention effectiveness. For many existing pathogens there are identified relationships between age and infection rates^30^. However, as we are modelling novel pathogens which could interact with age in unknown ways (biologically or behaviourally), as a simplification we assume that all ages have the same likelihood per contact of being infected and the same duration of exposure and infection.

### Interventions

#### Testing and contact tracing

Five combinations of testing, tracing, isolation and quarantine were modelled: testing and isolation (TI); testing, tracing and isolation (TTI); testing, tracing, isolation and quarantine of 7 days (TTIQ, Q = 7); and testing, tracing, isolation and quarantine of 14 days (TTIQ, Q = 14).

Symptomatic testing was parametrised by a single probability of testing if infectious-symptomatic that was assigned to agents on their first day of being infectious with a 2-day delay between symptom onset to testing (Table 1). Those who test positive enter isolation, where they remain for their entire infectious duration and have no further transmission. In scenarios with contract tracing, the contacts of an agent that tests positive can also trigger contact tracing, with their contacts having a network-specific probability of being traced and tested with one day delay (Table 1). In scenarios with quarantine, contacts that were traced enter quarantine for a scenario-specific duration, with no transmission taking place during the quarantine period.

#### Public health and social measures

The public health and social measures considered were: masks only, work from home advised, school closure, work from home advised and school closure, and lockdown. Each of these scenarios were modelled as changes in attendance and daily transmission probability in different networks (Table 1). In this study the term school closure is used to represent reduced attendance and risk reduction measures within schools with details described in Table 1.

### Pathogen characteristics

The pathogen characteristics varied for this analysis were daily transmission probability, latent period, infectious period and symptomatic fraction. As daily transmission probability does not have a direct biological analogue and is instead a model-specific parameter governing per-contact transmission, for each combination of disease parameters, a theoretical R_0_ was calculated. R_0_ was calculated by identifying agents in the model who were assigned the initial seed infections, and then calculating the average number of secondary infections resulting from these seed infections.

The pathogen characteristic ranges were chosen to cover a range of possible characteristics for airborne pathogens (Table 1). Each pathogen was generated by sampling each pathogen characteristic independently from uniform distributions.

### Outcomes and analysis

A collection of 5000 pathogens was generated covering possible combinations of R_0_, latent period, infectious period and symptomatic fraction (Table 1; Fig. 1). Simulations for each generated pathogen with no interventions were run for 1-year, ensuring each pathogen reached elimination (no new infections for over a 7-day period) before the end of the simulation timeframe and providing the baseline outcome. Then for each generated pathogen, simulations were run for all scenarios considered, with interventions in place for the entire simulation duration. Each simulation began with 25 infections randomly seeded into the model population, reflecting the assumption that interventions would likely be triggered at this point. The number of infections at 60 days and the total number of infections was recorded.

This analysis focuses on the early epidemic phase, when broad, non-pharmaceutical interventions are most likely to be deployed. A 60-day window was chosen as an approximation of this early phase, as beyond this point interventions would likely be tailored in response to more information about the pathogen. Total infections are additionally reported to provide context on the overall outbreak timescale and to distinguish between scenarios that delay transmission and those that substantially reduce cumulative spread.

To assess how latent period, infectious period and symptomatic fraction may impact the effectiveness of interventions, each of the 5000 pathogen samples were classified as above or below the median sampled value of each characteristic. For each disease characteristic, all samples above the median were considered high values, and all those below the median were considered low values. For each intervention scenario, the relative reduction in 60-day and total infections was compared for pathogens with low versus high values of each characteristic. Bootstrapping methods were used to calculate the mean percentage reduction in 60-day and total infections and 95% CIs of the means for each scenario compared to the scenario with no interventions. The reductions relative to no interventions are computed relative to the no intervention simulation with matching parameters.

## Results

### No interventions (baseline)

**Fig. 2:**
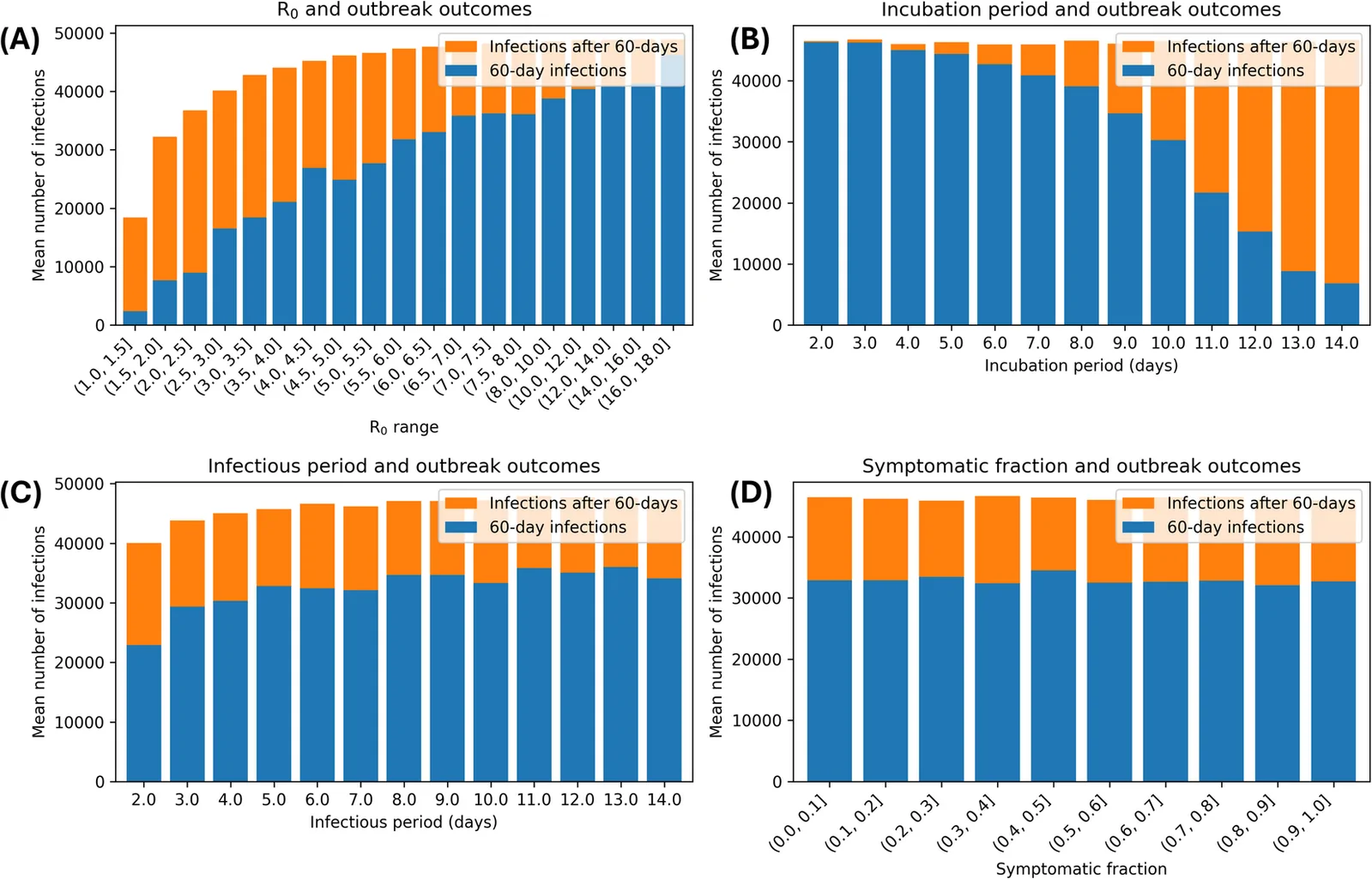
Simulation results with no interventions in place, orange corresponds to infections by 150 days and blue corresponds to infections after 60 days. Figure has simulation results for different (**A**) R_0_, (**B**) latent period, (**C**) infectious period and (**D**) symptomatic fraction ranges.

To understand the model dynamics before comparing intervention scenarios, Fig. 2 shows the impact of the different parameters on 60-day and total infections with no interventions in place. R_0_ is the main driver of total outbreak size, and also a major driver of the number of first 60-day infections, with the largest range in mean total infections (18,423 − 48,872) and mean 60-day infections (2,380 − 46,117) for the values studied. Latent period does not impact the total outbreak size but greatly impacts the first 60 days of an outbreak, with a small latent period resulting in a faster outbreak spread. With no interventions in place, the infectious duration and symptomatic fraction had minimal impact on the outbreak size. Timeseries of cumulative infections for different parameter ranges are shown in Figure A1 in the supplementary material.

The results shown in Fig. 2 are based on 4,800 simulations with R_0_ values ranging from 1 to 18. For the main analysis, we restrict to simulations with R_0_ between 2 and 7 to lessen the impact of R_0_ on the outcomes, leaving 1,800 simulations. The corresponding analysis across the full R_0_ range is provided in Appendix A.

### TTIQ

**Fig. 3:**
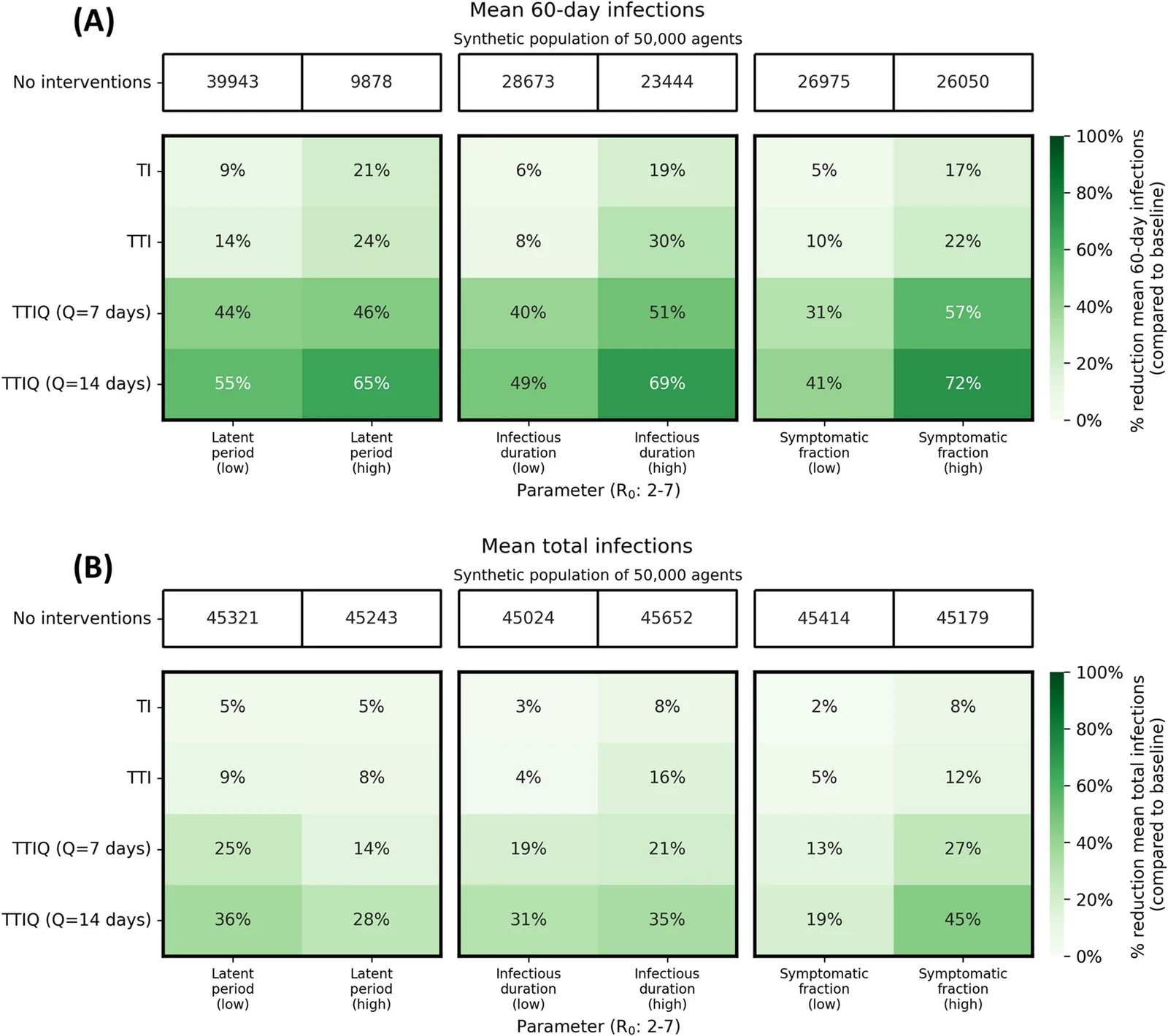
Relative impact of different test, trace, isolation and quarantine (TTIQ) interventions compared to no interventions for different pathogen characteristic ranges. Colour is used for illustrative purposes with light green meaning less infections averted, and dark green meaning more infections averted. (**A**) Relative impact on 60-day infections, (**B**) relative impact on total infections. Uncertainty ranges can be found in Table 2.

**Fig. 4:**
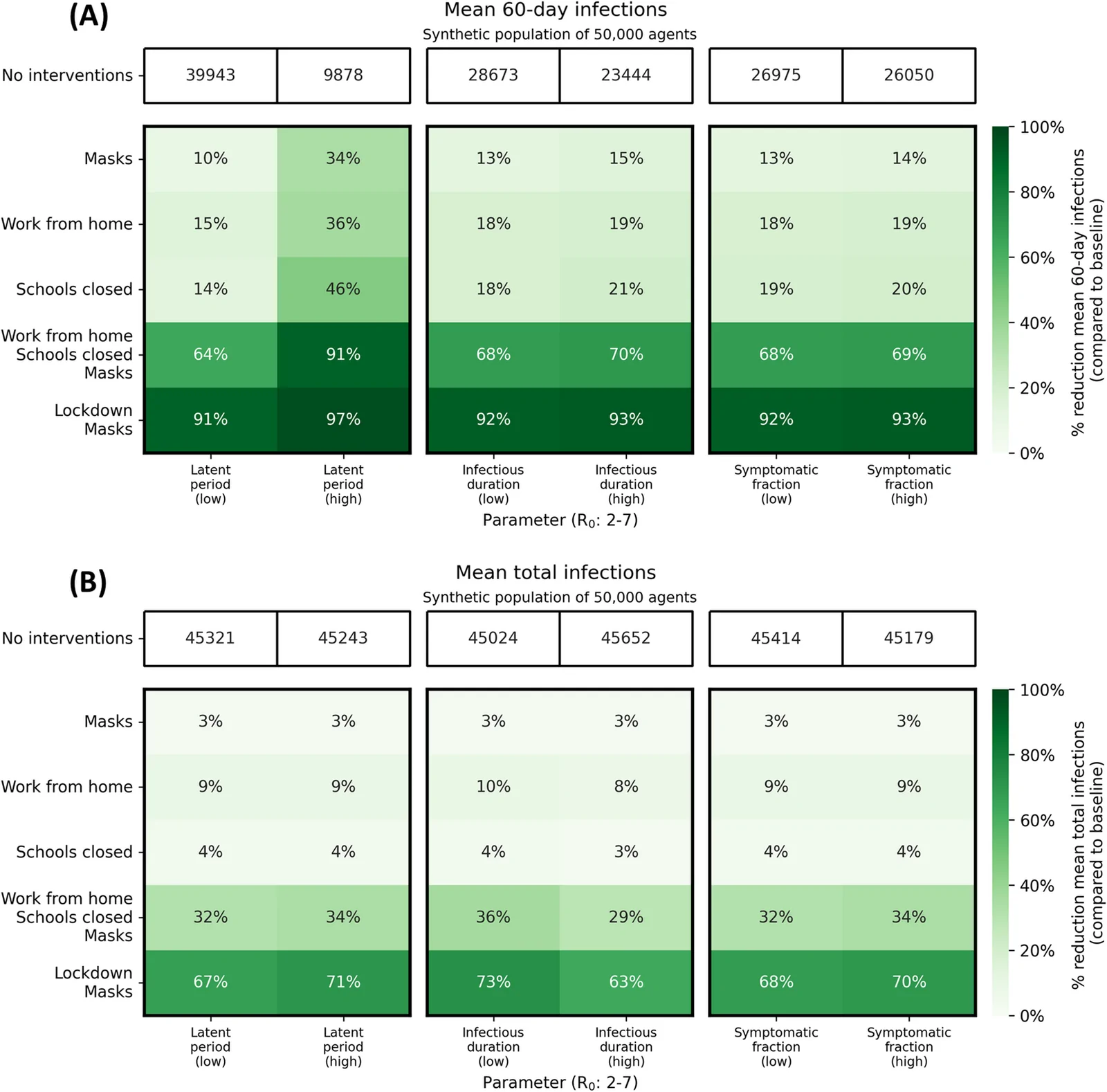
Impact of public health and social measures compared to no interventions for different disease parameter ranges. Colour is used for illustrative purposes with light green meaning less infections averted, and dark green meaning more infections averted. (**A**) Relative impact on 60-day infections, (**B**) relative impact on total infections. Uncertainty ranges can be found in Table 3.

Figures 3 and 4 show the percentage reduction in infections for each scenario, for the high/low ranges of each parameter. For each disease characteristic, the columns represent the 1,800 simulations split on either side of the median value with low (bottom 50-percentile) and high (top 50-percentile) each comprising 900 simulations. Scenarios for each parameter range are compared to no interventions with the same parameter range to calculate percentage reduction in infections.

Regardless of pathogen characteristics, any level of TTIQ reduced the number of 60-day infections (5–75%) and total infections (2–56%) compared to no interventions. The relative impact of TI and TTI scenarios was highest for pathogens with high infectious duration probability for both 60-day (19–73%) and total infections (8–47%) and for pathogens with a high symptomatic fraction for both 60-day (17–75%) and total infections (8–56%). Within the first 60 days, infectious duration was a bigger driver of the effectiveness of TI and TTI than symptomatic fraction. However, the relative impact of TI and TTI scenarios on total infections is equivalent for pathogens with high infections duration and high symptomatic fraction.

Introducing quarantine greatly increased the relative impact of contact tracing interventions for all pathogen characteristics.

**Table 2:** Relative impact with uncertainty of different TTIQ interventions compared to no interventions for 60-day and total infections. For each scenario, the mean is reported along with its 95% confidence interval (shown in brackets).

TTIQ	Metric	Latent period (low)	Latent period (high)	Infectious duration (low)	Infectious duration (high)	Symptomatic fraction (low)	Symptomatic fraction (high)
No interventions	60-day Infections	39,943 (39156, 40716)	9878 (9177, 10656)	28,673 (27520, 29747)	23,445 (22178, 24870)	26,975 (25735, 28164)	26,050 (24817, 27231)
TI	60-day Infections	9% (8%, 10%)	21% (19%, 23%)	6% (5%, 6%)	19% (18%, 21%)	5% (4%, 5%)	17% (15%, 18%)
TTI	60-day Infections	14% (13%, 15%)	24% (22%, 26%)	8% (7%, 9%)	30% (28%, 32%)	10% (9%, 11%)	22% (20%, 23%)
TTIQ (Q = 7 days)	60-day Infections	44% (42%, 45%)	46% (43%, 48%)	40% (38%, 41%)	51% (49%, 54%)	31% (29%, 32%)	57% (55%, 59%)
TTIQ (Q = 14 days)	60-day Infections	55% (53%, 57%)	65% (62%, 67%)	49% (48%, 51%)	69% (67%, 72%)	41% (40%, 43%)	72% (70%, 74%)
No interventions	Total Infections	45,322 (45103, 45521)	45,243 (44997, 45488)	45,024 (44793, 45261)	45,652 (45407, 45905)	45,415 (45201, 45654)	45,179 (44938, 45394)
TI	Total Infections	5% (4%, 5%)	5% (5%, 6%)	3% (2%, 3%)	8% (7%, 9%)	2% (2%, 2%)	8% (7%, 9%)
TTI	Total Infections	9% (9%, 10%)	8% (7%, 9%)	4% (4%, 4%)	16% (15%, 17%)	5% (5%, 5%)	12% (11%, 13%)
TTIQ (Q = 7 days)	Total Infections	25% (24%, 26%)	14% (14%, 15%)	19% (18%, 21%)	21% (20%, 22%)	13% (12%, 14%)	27% (26%, 28%)
TTIQ (Q = 14 days)	Total Infections	36% (35%, 37%)	28% (27%, 30%)	31% (29%, 32%)	35% (33%, 37%)	19% (19%, 20%)	45% (44%, 47%)

### Public health and social measures

Figure 4 shows the percentage reduction in infections for each PHSM scenario given a particular parameter range. Scenarios for each parameter range are compared to the respective infection numbers from the no intervention scenario.

Regardless of pathogen characteristics, any level of PHSM results in a decrease in the number of 60-day infections (10–97%) and total infections (3–73%) compared to no interventions. The relative impact of PHSMs was equivalent for pathogens with different infectious duration and symptomatic fractions, for both 60-day (13–93%) and total infections (3–73%) compared to the no interventions scenario.

In the first 60 days, masks and PHSM had a higher relative impact for pathogens with a high latent period (34–97%) compared to a low latent period (10–91%). A high latent period results in a delay of the peak number of daily infections, by introducing PHSM the peak is further delayed resulting in less infections in the first 60 days. However, when looking at total outbreak size, all PHSM had similar effect sizes regardless of pathogen characteristics as all outbreaks had ended.

Masks, work from home recommendations and school closures all had similar effectiveness on 60-day and total infections with the exception of pathogens with high latent periods. Furthermore, the coupling of work from home recommendations and school closures had a 2- to 3-fold increase in effect size on 60-day infections and 5-fold increase in effect size on total infections compared to work from home recommendations or school closures in isolation. Lockdowns had the greatest effect size, with a 2-fold increase and 3-fold increase in effect size on 60-day and total infections compared to the coupling of work from home recommendations and school closures respectively.

**Table 3:** Relative impact with uncertainty of different PHSM interventions compared to no interventions for 60-day and total infections. For each scenario, the mean is reported along with its 95% confidence interval (shown in brackets).

PHSM	Metric	Latent period (low)	Latent period (high)	Infectious duration (low)	Infectious duration (high)	Symptomatic fraction (low)	Symptomatic fraction (high)
No interventions	60-day Infections	39,943 (39156, 40716)	9878 (9177, 10656)	28,673 (27520, 29747)	23,445 (22178, 24870)	26,975 (25735, 28164)	26,050 (24817, 27231)
Masks	60-day Infections	10% (9%, 10%)	34% (32%, 35%)	13% (12%, 14%)	15% (14%, 16%)	13% (12%, 14%)	14% (13%, 15%)
Work from home advised	60-day Infections	15% (14%, 15%)	36% (35%, 37%)	18% (17%, 19%)	19% (17%, 20%)	18% (17%, 19%)	19% (18%, 20%)
Schools closed	60-day Infections	14% (13%, 15%)	46% (45%, 48%)	18% (17%, 20%)	21% (19%, 23%)	19% (17%, 20%)	20% (19%, 22%)
Schools closed & work from home advised + masks	60-day Infections	64% (62%, 66%)	91% (91%, 92%)	68% (65%, 70%)	70% (67%, 73%)	68% (65%, 70%)	69% (66%, 71%)
Lockdown + masks	60-day Infections	91% (90%, 93%)	97% (97%, 97%)	92% (91%, 93%)	93% (91%, 94%)	92% (91%, 93%)	93% (91%, 94%)
No interventions	Total Infections	45,322 (45103, 45521)	45,243 (44997, 45488)	45,024 (44793, 45261)	45,652 (45407, 45905)	45,415 (45201, 45654)	45,179 (44938, 45394)
Masks	Total Infections	3% (3%, 3%)	3% (3%, 3%)	3% (3%, 3%)	3% (2%, 3%)	3% (3%, 3%)	3% (3%, 3%)
Work from home advised	Total Infections	9% (9%, 9%)	9% (9%, 10%)	10% (9%, 10%)	8% (8%, 9%)	9% (8%, 9%)	9% (9%, 10%)
Schools closed	Total Infections	4% (3%, 4%)	4% (4%, 4%)	4% (4%, 4%)	3% (3%, 4%)	4% (3%, 4%)	4% (4%, 4%)
Schools closed & work from home advised + masks	Total Infections	32% (31%, 34%)	34% (32%, 36%)	36% (34%, 38%)	29% (27%, 31%)	32% (30%, 34%)	34% (32%, 36%)
Lockdown + masks	Total Infections	67% (66%, 69%)	71% (69%, 73%)	73% (72%, 75%)	63% (61%, 65%)	68% (66%, 70%)	70% (68%, 72%)

### R_0_ sensitivities

The same analysis is repeated for pathogens of different R_0_ ranges, and can be found in Appendix A. For smaller R_0_ values, a large latent period resulted in very small amounts of 60-day infections, meaning the impact of scenarios appeared minimal as it was impossible for the scenarios to reduce the number of seeded infections. For higher R_0_ values, the relationships between pathogen characteristics and interventions were similar given an R_0_ of 2–7, just with reduced relative impact.

Specific subsets of parameters reflecting more influenza-like, COVID-19-like, chickenpox-like and measles-like diseases are explored in Appendix A.6.

## Discussion

This analysis used an agent-based model to simulate outbreaks of novel pathogens with different characteristics and determine the effectiveness of different intervention combinations in reducing infections in the first 60 days before a more tailored response would be implemented. The study compared the effectiveness of interventions both in the first 60 days as well as at the end of an outbreak, to investigate interventions that simply delay the peak compared to full outbreak containment. In this study, R_0_ was the strongest determinant of total outbreak size and latent period is the strongest determinant on the number of infections in the first 60 days. We found that TTIQ is likely to have the greatest relative impact for pathogens with higher infectious duration or symptomatic fraction, however PHSM are likely to be equivalent for most pathogen characteristics in terms of total infections averted.

With no interventions in place, R_0_ was the only determinant, among those tested, of total outbreak size thereby highlighting the impact R_0_ has on outbreak dynamics. This is consistent with previous modelling studies that have demonstrated the importance of knowing R_0_ to predict epidemic spread^24^. When a new pathogen emerges R_0_ is usually one of the first characteristics to be estimated, as it is well known, has a strong relationship with total outbreak size, and there are clear procedures on how to measure it^31,32^. Extending previous work, we also found that pathogens with similar R_0_ may have very different characteristics such as latent period, infectious duration and symptomatic fraction, which may impact the speed of the outbreak or the relative effectiveness of interventions. Therefore, while the importance of R_0_ should not be understated, learning about other pathogen characteristics is critical to inform which packages of interventions should be implemented, how quickly they need to be put in place, and how long they may need to be in place for.

While R_0_ was critical for estimating total outbreak size, the latent period of a pathogen was the strongest determinant of infections in the first 60 days of an outbreak. For a high latent period, the outbreak spread was slower and there were fewer infections in the first 60 days compared to pathogens with the same R_0_ but shorter latent periods. This could influence how fast governments and policy makers must determine suitable interventions and control strategies, as well as how long interventions may need to be in place for and hence whether different response types are likely to be considered proportional by the community. A pathogen with a high R_0_ but long latent period could also be inadvertently underestimated, for example if R_0_ was high but very few infectious were detected over the first 60 days, authorities may underestimate the potential of the pathogen for infecting large proportions of the population. By knowing the latent period of a disease in the early stages of a pathogen, it is possible to anticipate the speed of an outbreak in terms of new infections per day.

During the COVID-19 pandemic, testing, tracing, and isolation of positive cases was one of the early interventions introduced^8^. However, our modelling suggests that TTIQ interventions may not be as effective for all pathogen types, particularly those with short infectious periods or low symptomatic fractions. We assumed that 75% of symptomatic cases were tested, with a 2-day delay from symptom onset^33,34^. Symptomatic testing was more effective for pathogens with longer infectious periods, as diagnosed individuals were isolated for a greater proportion of their infectious period, thereby averting more transmission. In contrast, pathogens with shorter infectious periods result in less community infections making isolation less impactful. Interventions such as symptomatic testing, which kick-start TTIQ cycles, will be highly influenced by pathogen characteristics that reduce the likelihood of people self-testing. The infectious duration of a disease is usually estimated soon after its emergence, however, symptomatic fraction is harder to ascertain and usually has great amounts of uncertainty surrounding it (from early COVID-19 estimates: 5–80% of cases were potentially asymptomatic)^35,36^. Without knowing a more precise range of values for symptomatic fraction, interventions that include asymptomatic testing (outside of contact tracing), such as regular screening for workplaces and schools, may have a role to help estimate the pathogen characteristics and to improve the effectiveness of TTIQ.

An early COVID-19 intervention was 14-day quarantine for people who were close contact of a confirmed COVID-19 case^11^. The results from this study show that any length of quarantine could greatly improve outcomes in the first 60 days of an outbreak, irrespective of pathogen characteristics. For the pathogens tested, the latent period was sampled between 2 and 14 days, however if a future pathogen had a longer latent period, potentially a longer quarantine duration would be needed. Learning about a disease latent period could be used to inform quarantine durations, and aid to avoid the use of unnecessarily long quarantine periods. Furthermore, unnecessarily long quarantine periods could result in reduced symptomatic testing or compliance with contract tracing due to hesitancy towards extended school or work absenteeism.

Our modelling suggests that latent period influenced the relative impact of PHSM in the first 60 days of an outbreak, but not total outbreak size. In general, all PHSM tested were effective at reducing infections. On emergence of a future pathogen, likely no disease characteristics will have been identified. It is possible to use PHSM to slow the initial spread of the disease, allowing more time to research pathogen characteristics. Once characteristics have been identified, it is then possible to introduce more tailored responses best suited to the disease such as testing and contact tracing. In the long-term PHSM will likely need to be chosen irrespective of disease characteristics, with the nuance around what interventions will have the best public health outcomes while also not being overly restrictive.

This analysis focused on the first 60 days of an emergent pathogen, on the basis that beyond this period more bespoke analyses would be possible and more detailed overall strategies could be developed. For future pandemics it will be integral to have an overall guiding strategy that considers responses that are proportional, best protect those most vulnerable, and are tailored to the characteristics of the emerging disease. Early decisions will need to be made around whether the goals are for total disease eradication, or a more long-term plan to minimise ongoing infections in the community^37^. Disease characteristics relating to severity, such as mortality rate and long-term consequences, will likely influence what goals are chosen. This study did not investigate these different goals, but rather the interactions between pathogen characteristics and the relative effectiveness of potential interventions that could buy time while these decisions are being made. For example, by delaying an early peak in infections, it could be possible to gain time to make informed decisions regarding pandemic preparedness strategies.

There are some limitations to this study. First, this is a theoretical analysis, and it is not intended as a predictor of outbreaks of future pathogens but instead is used to test effectiveness of different interventions on different pathogens. Second, we used previous COVID-19 modelling studies to inform the efficacy of different interventions (see Table 1), but as there may be less social license for disease control interventions their efficacy may be overestimated in this study. However, the conclusions regarding the interaction between policies and disease characteristics will still be the same, just with a lower effect size. Third, demographic data and intervention effectiveness were drawn from previous COVID-19 studies done in collaboration with the Victorian government^38,39^. Demographic data and intervention effectiveness will differ by country, which will impact the intervention effect sizes seen in this analysis. Fourth, the study assumed all interventions were in place upon emergence of the future pathogen, which is optimistic and may overestimate the intervention effect sizes. Different seeding assumptions, such as number and timing of initial cases, would affect the baseline number of infections, but because interventions act as proportional reductions in transmission, relative reductions in infections are expected to be broadly consistent. Fifth, the model does not consider pathogens with pre-symptomatic transmission (i.e. transmission before an agent has entered the infectious-symptomatic stage of the disease). TTIQ interventions would be less effective for pathogens with presymptomatic transmission (see Appendix A.7). Sixth, a range of likely values for latent period and infectious duration were chosen based on previous studies around respiratory viruses^40^. For pathogens with longer latent or infectious periods, a higher quarantine period might be needed to see the optimal effects. Seventh, testing was assumed to be instantaneous, with perfect sensitivity and specificity and complete isolation of all positive cases. Lower test sensitivity could be approximated by modelling a pathogen with a smaller symptomatic fraction, which would decrease the effectiveness of TTIQ. Eight, this study assumed the same transmission risk for all ages, when diseases such as influenza traditionally show the highest attack rates among children^30^. This simplification may result in less representative effect sizes for PHSM such as work from home recommendations and school closures. Lastly, severity has been excluded from this study; hence results should be interpreted as a disease that is severe enough to warrant a response.

## Conclusion

Determining pathogen characteristics in the early days of an outbreak could be highly beneficial for policy makers when selecting interventions for better health outcomes in the community. Identifying a pathogen’s R_0_ can inform the total expected outbreak size, its latent period can inform the speed of the outbreak, time to respond and expected duration a response will be required for, and its infectious duration and symptomatic fraction can inform the relative effectiveness of intervention packages. By determining disease characteristics early, interventions could be put in place to delay the peak in infections, hence giving policy makers more time to determine long-term optimal strategies for pandemic control with the best interests of public health at the forefront.

## Supplementary Information

Below is the link to the electronic supplementary material.

Supplementary Material 1

## Data Availability

The code used for this analysis and datasets that were generated are available in Zenodo: https://doi.org/10.5281/zenodo.18453165 (3 Feb 2026).

## References

[1] Sampath, S. et al. *Pandemics Throughout History Cureus*, **13**(9): e18136. (2021). 10.7759/cureus.18136 PMC8525686 34692344

[2] Gavi *5 Reasons Why Pandemics Like COVID-19 Are Becoming More Likely*. (2020).

[3] Feng, Z. et al. Timely identification of optimal control strategies for emerging infectious diseases. *J. Theor. Biol.* **259** (1), 165–171 (2009). 10.1016/j.jtbi.2009.03.006 PMC7126620 19289133

[4] Nishiura, H. Correcting the actual reproduction number: a simple method to estimate R(0) from early epidemic growth data. *Int. J. Environ. Res. Public. Health*. **7** (1), 291–302 (2010). 10.3390/ijerph7010291 PMC2819789 20195446

[5] Goldstein, E. et al. Reproductive numbers, epidemic spread and control in a community of households. *Math. Biosci.* **221** (1), 11–25 (2009). 10.1016/j.mbs.2009.06.002 PMC2731010 19559715

[6] Schneiders, M. L. et al. The impact of COVID-19 non-pharmaceutical interventions on the lived experiences of people living in Thailand, Malaysia, Italy and the United Kingdom: A cross-country qualitative study. *PLoS One*. **17** (1), e0262421 (2022). 10.1371/journal.pone.0262421 PMC8782407 35061789

[7] World Health Organisation, Coronavirus disease (COVID-19) travel advice, in Key considerations for repatriation and quarantine of travellers in relation to the outbreak of novel coronavirus 2019-nCoV. : (2020). https://www.who.int/news-room/articles-detail/key-considerations-for-repatriation-and-quarantine-of-travellers-in-relation-to-the-outbreak-of-novel-coronavirus-2019-ncov

[8] World Health Organisation. *Timeline: WHO’s COVID-19 response*. Coronavirus disease (COVID-19) 2025 2025].

[9] National Academies of Sciences, E., and, Health, M. & Medicine *Implementing Quarantine to Reduce or Stop the Spread of a Contagious Disease*, in *Evidence-Based Practice for Public Health Emergency Preparedness and Response.*, A. Downey, L. Brown, and N. Calonge, Editors. National Academies Press: (2020). https://www.ncbi.nlm.nih.gov/books/NBK563999/33211452

[10] Jiang, X. et al. *Is a 14-day quarantine period optimal for effectively controlling coronavirus disease 2019 (COVID-19)?* : p. 2020.03. 15.20036533. (2020).

[11] World Health Organization. *Considerations for quarantine of contacts of COVID-19 cases: interim guidance, 19 August 2020*, in *Considerations for quarantine of contacts of COVID-19 cases: interim guidance, 19 August 2020*. (2020).

[12] Chen, T. *Reducing COVID-19 transmission through cleaning and disinfecting household surfaces* p. 1–18 (National Collaborating Centre for Environmental Health, 2020).

[13] Eikenberry, S. E. et al. To mask or not to mask: Modeling the potential for face mask use by the general public to curtail the COVID-19 pandemic. *Infect. Dis. Model.* **5**, 293–308 (2020). 10.1016/j.idm.2020.04.001 PMC7186508 32355904

[14] Lewis, D. *COVID-19 rarely spreads through surfaces. So why are we still deep cleaning?* Nature: (2021). https://www.nature.com/articles/d41586-021-00251-4. p. 590, 26–28. 10.1038/d41586-021-00251-4 33514939

[15] Haug, N. et al. Ranking the effectiveness of worldwide COVID-19 government interventions. *Nat. Hum. Behav.* **4** (12), 1303–1312 (2020). 10.1038/s41562-020-01009-0 33199859

[16] Mendez-Brito, A., Bcheraoui, C. E. & Pozo-Martin, F. Systematic review of empirical studies comparing the effectiveness of non-pharmaceutical interventions against COVID-19. *J. Infect.* **83** (3), 281–293 (2021). 10.1016/j.jinf.2021.06.018 PMC8214911 34161818

[17] Coccia, M. Different effects of lockdown on public health and economy of countries: Results from first wave of the COVID-19 pandemic. *J. Econ. Libr.* **8** (1), 45–63 (2021).

[18] Fraser, C. et al. *Factors that make Infect. disease outbreak controllable* **101**(16): 6146–6151. (2004). 10.1073/pnas.0307506101 PMC395937 15071187

[19] Kermack, W. O. & McKendrick, A. G. *contribution Math. theory epidemics* **115**(772): 700–721. (1927).

[20] Brauer, F. Compartmental models in epidemiology. *Mathematical epidemiology.* 19-79 (2008).

[21] Arsal, S. R., Aldila, D. & Handari, B. D. *Short review of mathematical model of measles*. in *AIP Conference Proceedings*. AIP Publishing. (2020).

[22] Osthus, D. et al. Forecasting seasonal influenza with a state-space SIR model. *Ann. Appl. Stat.* **11** (1), 202–224 (2017). 10.1214/16-AOAS1000 PMC5623938 28979611

[23] He, S., Peng, Y. & Sun, K. SEIR modeling of the COVID-19 and its dynamics. *Nonlinear Dyn.* **101** (3), 1667–1680 (2020). 10.1007/s11071-020-05743-y PMC7301771 32836803

[24] van den Driessche, P. & Watmough, J. Reproduction numbers and sub-threshold endemic equilibria for compartmental models of disease transmission. *Math. Biosci.* **180**, 29–48 (2002). 10.1016/s0025-5564(02)00108-6 12387915

[25] Rozán, E. A. et al. *importance incubation time distribution compartmental Epidemiol. models* **650**: 129998. (2024).

[26] Nunes, M. C. et al. Redefining pandemic preparedness: Multidisciplinary insights from the CERP modelling workshop in infectious diseases, workshop report. *Infect. Dis. Model.* **9** (2), 501–518 (2024). 10.1016/j.idm.2024.02.008 PMC10912817 38445252

[27] Wu, J. T. & Cowling, B. J. The use of mathematical models to inform influenza pandemic preparedness and response. *Exp. Biol. Med. (Maywood)*. **236** (8), 955–961 (2011). 10.1258/ebm.2010.010271 PMC3178755 21727183

[28] Institute for Disease Modelling. Starsim model GitHub repository. (2024). Available from: https://github.com/starsimhub/starsim

[29] Delport, D. et al. *Estimating the historical impact of outbreak response immunisation programmes across 210 outbreaks in low and middle-income countries*. *BMJ Glob Health*, **10**(7), (2025). 10.1136/bmjgh-2024-016887 PMC12258375 40633967

[30] Munoz, F. M. The impact of influenza in children. *Semin Pediatr. Infect. Dis.* **13** (2), 72–78 (2002). 10.1053/spid.2002.122992 12122955

[31] Dietz, K. The estimation of the basic reproduction number for infectious diseases. *Stat. Methods Med. Res.* **2** (1), 23–41 (1993). 10.1177/096228029300200103 8261248

[32] Kwok, K. O. et al. Early real-time estimation of the basic reproduction number of emerging or reemerging infectious diseases in a community with heterogeneous contact pattern: Using data from Hong Kong 2009 H1N1 Pandemic Influenza as an illustrative example. *PLoS One*. **10** (9), e0137959 (2015). 10.1371/journal.pone.0137959 PMC4570805 26372219

[33] Hellard, M. et al. *The Optimise Study: Summer 2021–2022 Snapshot.*, in *The Optimise Study*. (2022). https://www.burnet.edu.au/media/1kthghrv/optimise_snapshot_summer_final.pdf

[34] Office for National Statistics (ONS). *Coronavirus (COVID-19) testing behaviours and outcomes, England: September 2020 to November 2021.* (2022). https://www.ons.gov.uk/releases/coronaviruscovid19testingbehavioursandoutcomesbysociodemographiccharacteristicsenglandseptember2020tonovember2021

[35] Heneghan, C., Brassey, J. & Jefferson, T. *COVID-19: What proportion are asymptomatic?* Evidence Service to support the COVID-19 response 2020 2 July 2025].

[36] Byambasuren, O. et al. Estimating the extent of asymptomatic COVID-19 and its potential for community transmission: Systematic review and meta-analysis. *J. Assoc. Med. Microbiol. Infect. Dis. Can.* **5** (4), 223–234 (2020). 10.3138/jammi-2020-0030 PMC9602871 36340059

[37] Holmberg, M. & Lundgren, B. Framing post-pandemic preparedness: Comparing eight European plans. *Glob Public. Health*. **13** (1), 99–114 (2018). 10.1080/17441692.2016.1149202 26948258

[38] Scott, N. et al. COVID-19 epidemic modelling for policy decision support in Victoria, Australia 2020–2021. *BMC Public. Health*. **23** (1), 988 (2023). 10.1186/s12889-023-15936-w PMC10219801 37237343

[39] Australian Bureau of Statistics, Labour Force, Australia, Detailed, in ABS. : (2021). https://www.abs.gov.au/statistics/labour/employment-and-unemployment/labour-force-australia-detailed/latest-release#data-download

[40] Lessler, J. et al. Incubation periods of acute respiratory viral infections: a systematic review. *Lancet Infect. Dis.* **9** (5), 291–300 (2009). 10.1016/S1473-3099(09)70069-6 PMC4327893 19393959

