# Supplementary material for: The effectiveness of outbreak response measures for pathogens of different characteristics
Source: Sci Rep. 2026 Aug 10;16:37436. doi: 10.1038/s41598-026-64138-y (PMC13747814; doi:10.1038/s41598-026-64138-y)
Supplement: Supplementary file 1 [file 41598_2026_64138_MOESM1_ESM.docx]

Supplementary Material

Appendix A Additional results
           A.1 Baseline

Figure A1 shows the cumulative infections from the modelled simulations across different parameter ranges. The R₀ affects both the speed and the final size of the outbreak, with higher R₀ values leading to faster growth and larger total numbers of infections. The latent period influences the speed of the outbreak, with longer latent periods delaying epidemic growth, but has little impact on the final epidemic size. The infectious duration affects both the speed and final size of the outbreak, although its influence is less pronounced than that of R₀.

Figure A1: Cumulative infections over time for different parameter ranges.

A.2 R0 = 1-2
    A.2.1 TTIQ

TTIQ scenarios were more effective for pathogens with a greater infectious duration and symptomatic fraction (Figure A2, Table A1). TTIQ scenarios were less effective for scenarios with a higher latent period. This is due to the small number of infections in the first 60-days, meaning the number of initially infected individuals resulting in edge effects. For total infections, TTIQ scenarios were more effective for pathogens with a high infectious duration and symptomatic fraction.

Figure A2: Relative impact of different test, trace, isolation and quarantine (TTIQ) interventions compared to no interventions for different pathogen characteristic ranges. Colour is used for illustrative purposes with light green meaning less infections averted, and dark green meaning more infections averted. (A) Impact on 60-day infections, (B) impact on total infections.

Table A1: Relative impact with uncertainty of different TTIQ interventions compared to no interventions for 60-day and total infections.

| TTIQ_label | Metric | Latent period (low) | Latent period (high) | Infectious duration (low) | Infectious duration (high) | Symptomatic fraction (low) | Symptomatic fraction (high) |
| --- | --- | --- | --- | --- | --- | --- | --- |
| No interventions | 60-day Infections | 9171 (6945, 11452) | 423 (325, 547) | 7292 (5425, 9425) | 2621 (1638, 3707) | 5571 (3726, 7503) | 4842 (3299, 6585) |
| TI | 60-day Infections | 27% (20%, 36%) | 26% (20%, 33%) | 17% (11%, 24%) | 62% (46%, 75%) | 15% (8%, 26%) | 40% (30%, 54%) |
| TTI | 60-day Infections | 34% (26%, 45%) | 27% (20%, 33%) | 22% (16%, 29%) | 77% (64%, 86%) | 25% (15%, 38%) | 44% (34%, 60%) |
| TTIQ (Q=7 days) | 60-day Infections | 75% (69%, 82%) | 44% (36%, 55%) | 72% (63%, 80%) | 82% (72%, 91%) | 60% (51%, 68%) | 90% (84%, 94%) |
| TTIQ (Q=14 days) | 60-day Infections | 79% (73%, 85%) | 56% (49%, 66%) | 76% (68%, 83%) | 85% (76%, 92%) | 65% (56%, 72%) | 92% (87%, 96%) |
| No interventions | Total Infections | 26699 (24838, 28686) | 24795 (22276, 27239) | 26554 (24857, 28317) | 25029 (22716, 27172) | 26218 (24108, 28225) | 25564 (23605, 27739) |
| TI | Total Infections | 26% (21%, 31%) | 29% (22%, 36%) | 15% (12%, 19%) | 43% (37%, 50%) | 13% (10%, 16%) | 41% (35%, 49%) |
| TTI | Total Infections | 35% (29%, 41%) | 33% (26%, 41%) | 18% (14%, 22%) | 57% (50%, 64%) | 20% (16%, 24%) | 48% (41%, 57%) |
| TTIQ (Q=7 days) | Total Infections | 65% (59%, 72%) | 49% (41%, 57%) | 53% (47%, 58%) | 66% (59%, 73%) | 38% (33%, 42%) | 79% (74%, 84%) |
| TTIQ (Q=14 days) | Total Infections | 72% (66%, 79%) | 73% (65%, 80%) | 70% (64%, 76%) | 76% (69%, 83%) | 52% (46%, 58%) | 94% (91%, 96%) |

A.2.2 PHSM

PHSM scenarios had similar relative reductions in 60-day infections irrespective of infectious duration and symptomatic fraction (Figure A3, Table A2). Similar to the TTIQ scenarios, the PHSM scenarios in first 60-days were less effective on pathogens with a higher latent period. This again is due to the small number of infections in the first 60-days, meaning the number of initially infected individuals resulting in edge effects. For total infections, PHSM were not effected by pathogen characteristics.

Figure A3: Impact of public health and social measures compared to no interventions for different disease parameter ranges. Colour is used for illustrative purposes with light green meaning less infections averted, and dark green meaning more infections averted. (A) Impact on 60-day infections, (B) impact on total infections.

Table A2: Relative impact with uncertainty of different PHSM interventions compared to no interventions for 60-day and total infections.

| PHSM | Metric | Latent period (low) | Latent period (high) | Infectious duration (low) | Infectious duration (high) | Symptomatic fraction (low) | Symptomatic fraction (high) |
| --- | --- | --- | --- | --- | --- | --- | --- |
| No interventions | 60-day Infections | 9171 (6945, 11452) | 423 (325, 547) | 7292 (5425, 9425) | 2621 (1638, 3707) | 5571 (3726, 7503) | 4842 (3299, 6585) |
| Masks | 60-day Infections | 48% (41%, 56%) | 43% (40%, 46%) | 43% (35%, 52%) | 65% (60%, 69%) | 42% (33%, 54%) | 54% (43%, 65%) |
| Work from home advised | 60-day Infections | 47% (43%, 50%) | 42% (39%, 46%) | 44% (40%, 49%) | 54% (51%, 58%) | 45% (40%, 51%) | 48% (44%, 53%) |
| Schools closed | 60-day Infections | 60% (52%, 67%) | 46% (41%, 50%) | 55% (47%, 64%) | 74% (70%, 77%) | 55% (45%, 66%) | 64% (53%, 75%) |
| Schools closed & work from home advised + masks | 60-day Infections | 99% (99%, 99%) | 83% (80%, 87%) | 99% (98%, 99%) | 97% (96%, 98%) | 98% (98%, 99%) | 98% (98%, 99%) |
| Lockdown + masks | 60-day Infections | 99% (99%, 99%) | 86% (83%, 89%) | 99% (99%, 99%) | 98% (96%, 98%) | 99% (98%, 99%) | 99% (98%, 99%) |
| No interventions | Total Infections | 26699 (24838, 28686) | 24795 (22276, 27239) | 26554 (24857, 28317) | 25029 (22716, 27172) | 26218 (24108, 28225) | 25564 (23605, 27739) |
| Masks | Total Infections | 25% (23%, 29%) | 29% (25%, 34%) | 27% (24%, 30%) | 28% (24%, 32%) | 27% (23%, 30%) | 28% (25%, 31%) |
| Work from home advised | Total Infections | 39% (37%, 41%) | 40% (38%, 43%) | 39% (37%, 41%) | 40% (38%, 43%) | 40% (38%, 42%) | 40% (38%, 42%) |
| Schools closed | Total Infections | 33% (29%, 37%) | 38% (33%, 44%) | 35% (31%, 39%) | 36% (31%, 41%) | 34% (30%, 38%) | 37% (33%, 42%) |
| Schools closed & work from home advised + masks | Total Infections | 99% (97%, 100%) | 99% (97%, 100%) | 98% (96%, 100%) | 100% (99%, 100%) | 99% (98%, 100%) | 98% (97%, 100%) |
| Lockdown + masks | Total Infections | 100% (100%, 100%) | 100% (100%, 100%) | 100% (100%, 100%) | 100% (100%, 100%) | 100% (100%, 100%) | 100% (100%, 100%) |

A.3 R0 = 2-7
    A.3.1 TTIQ

Results can be found in the main paper.

A.3.2 PHSM

Results can be found in the main paper.

A.4 R0 = 7-12
    A.4.1 TTIQ

The results regarding the relationship between TTIQ effectiveness given pathogen characteristics is similar for R0 of 7-12 and a R0 of 2-7, but with smaller reductions in 60-day and total infections compared to the no interventions scenario.

Figure A4: Relative impact of different test, trace, isolation and quarantine (TTIQ) interventions compared to no interventions for different pathogen characteristic ranges. Colour is used for illustrative purposes with light green meaning less infections averted, and dark green meaning more infections averted. (A) Impact on 60-day infections, (B) impact on total infections.

Table A5: Relative impact with uncertainty of different TTIQ interventions compared to no interventions for 60-day and total infections.

| TTIQ_label | Metric | Latent period (low) | Latent period (high) | Infectious duration (low) | Infectious duration (high) | Symptomatic fraction (low) | Symptomatic fraction (high) |
| --- | --- | --- | --- | --- | --- | --- | --- |
| No interventions | 60-day Infections | 48337 (48267, 48406) | 27623 (26802, 28519) | 39022 (38303, 39724) | 38399 (37507, 39289) | 38807 (37997, 39611) | 38659 (37873, 39449) |
| TI | 60-day Infections | 2% (1%, 2%) | 15% (14%, 16%) | 5% (4%, 5%) | 7% (6%, 8%) | 3% (2%, 3%) | 9% (8%, 10%) |
| TTI | 60-day Infections | 4% (3%, 4%) | 18% (17%, 19%) | 7% (6%, 7%) | 11% (10%, 12%) | 5% (5%, 6%) | 12% (11%, 13%) |
| TTIQ (Q=7 days) | 60-day Infections | 14% (13%, 15%) | 34% (33%, 35%) | 20% (19%, 20%) | 22% (21%, 23%) | 15% (14%, 15%) | 26% (25%, 27%) |
| TTIQ (Q=14 days) | 60-day Infections | 30% (29%, 32%) | 55% (53%, 56%) | 38% (36%, 39%) | 39% (38%, 41%) | 27% (26%, 28%) | 50% (48%, 51%) |
| No interventions | Total Infections | 48566 (48547, 48583) | 48567 (48546, 48586) | 48466 (48446, 48485) | 48687 (48670, 48702) | 48573 (48553, 48591) | 48560 (48540, 48577) |
| TI | Total Infections | 1% (1%, 1%) | 1% (1%, 1%) | 1% (1%, 1%) | 1% (1%, 1%) | 0% (0%, 0%) | 2% (1%, 2%) |
| TTI | Total Infections | 3% (3%, 3%) | 2% (2%, 2%) | 2% (2%, 2%) | 3% (3%, 4%) | 2% (2%, 2%) | 3% (3%, 3%) |
| TTIQ (Q=7 days) | Total Infections | 8% (8%, 8%) | 4% (4%, 4%) | 7% (7%, 7%) | 6% (5%, 6%) | 5% (5%, 5%) | 8% (7%, 8%) |
| TTIQ (Q=14 days) | Total Infections | 17% (16%, 17%) | 9% (9%, 9%) | 15% (14%, 15%) | 11% (11%, 12%) | 9% (9%, 9%) | 17% (16%, 18%) |

A.4.2 PHSM

The results regarding the relationship between PHSM effectiveness given pathogen characteristics is similar for R0 of 7-12 and a R0 of 2-7, but with smaller reductions in 60-day and total infections compared to the no interventions scenario.

Figure A5: Impact of public health and social measures compared to no interventions for different disease parameter ranges. Colour is used for illustrative purposes with light green meaning less infections averted, and dark green meaning more infections averted. (A) Impact on 60-day infections, (B) impact on total infections.

Table A6: Relative impact with uncertainty of different PHSM interventions compared to no interventions for 60-day and total infections.

| PHSM | Metric | Latent period (low) | Latent period (high) | Infectious duration (low) | Infectious duration (high) | Symptomatic fraction (low) | Symptomatic fraction (high) |
| --- | --- | --- | --- | --- | --- | --- | --- |
| No interventions | 60-day Infections | 48337 (48267, 48406) | 27623 (26802, 28519) | 39022 (38303, 39724) | 38399 (37507, 39289) | 38807 (37997, 39611) | 38659 (37873, 39449) |
| Masks | 60-day Infections | 1% (1%, 1%) | 19% (18%, 20%) | 7% (7%, 8%) | 7% (7%, 8%) | 7% (7%, 8%) | 7% (7%, 8%) |
| Work from home advised | 60-day Infections | 2% (2%, 3%) | 20% (19%, 21%) | 8% (8%, 9%) | 8% (7%, 9%) | 8% (8%, 9%) | 8% (8%, 9%) |
| Schools closed | 60-day Infections | 2% (1%, 2%) | 28% (27%, 29%) | 11% (10%, 11%) | 10% (9%, 11%) | 10% (9%, 11%) | 10% (10%, 11%) |
| Schools closed & work from home advised + masks | 60-day Infections | 16% (15%, 17%) | 75% (74%, 76%) | 36% (35%, 38%) | 34% (32%, 36%) | 36% (34%, 38%) | 35% (33%, 37%) |
| Lockdown + masks | 60-day Infections | 49% (48%, 51%) | 94% (94%, 95%) | 66% (64%, 68%) | 62% (60%, 64%) | 65% (63%, 67%) | 64% (62%, 66%) |
| No interventions | Total Infections | 48566 (48547, 48583) | 48567 (48546, 48586) | 48466 (48446, 48485) | 48687 (48670, 48702) | 48572 (48553, 48591) | 48560 (48540, 48577) |
| Masks | Total Infections | 1% (1%, 1%) | 1% (1%, 1%) | 1% (1%, 1%) | 0% (0%, 0%) | 1% (1%, 1%) | 1% (1%, 1%) |
| Work from home advised | Total Infections | 1% (1%, 1%) | 1% (1%, 1%) | 2% (2%, 2%) | 1% (1%, 1%) | 1% (1%, 1%) | 1% (1%, 1%) |
| Schools closed | Total Infections | 0% (0%, 0%) | 0% (0%, 0%) | 0% (0%, 0%) | 0% (0%, 0%) | 0% (0%, 0%) | 0% (0%, 0%) |
| Schools closed & work from home advised + masks | Total Infections | 4% (4%, 4%) | 4% (4%, 4%) | 5% (4%, 5%) | 3% (3%, 3%) | 4% (4%, 4%) | 4% (4%, 4%) |
| Lockdown + masks | Total Infections | 14% (14%, 15%) | 14% (14%, 15%) | 18% (17%, 18%) | 10% (10%, 11%) | 14% (14%, 15%) | 15% (14%, 15%) |

A.5 R0 = 12-18
    A.5.1 TTIQ

The results regarding the relationship between TTIQ effectiveness given pathogen characteristics is similar for R0 of 12-18 and a R0 of 2-7, but with smaller reductions in 60-day and total infections compared to the no interventions scenario.

Figure A6: Relative impact of different test, trace, isolation and quarantine (TTIQ) interventions compared to no interventions for different pathogen characteristic ranges. Colour is used for illustrative purposes with light green meaning less infections averted, and dark green meaning more infections averted. (A) Impact on 60-day infections, (B) impact on total infections.

Table A7: Relative impact with uncertainty of different TTIQ interventions compared to no interventions for 60-day and total infections.

| TTIQ_label | Metric | Latent period (low) | Latent period (high) | Infectious duration (low) | Infectious duration (high) | Symptomatic fraction (low) | Symptomatic fraction (high) |
| --- | --- | --- | --- | --- | --- | --- | --- |
| No interventions | 60-day Infections | 48828 (48817, 48840) | 33440 (31768, 35031) | 41972 (40371, 43340) | 40394 (38837, 42061) | 41761 (40168, 43202) | 40681 (39051, 42393) |
| TI | 60-day Infections | 1% (0%, 1%) | 12% (11%, 14%) | 4% (3%, 5%) | 6% (5%, 8%) | 2% (2%, 3%) | 8% (7%, 10%) |
| TTI | 60-day Infections | 2% (2%, 2%) | 16% (14%, 18%) | 6% (5%, 7%) | 9% (8%, 11%) | 4% (3%, 5%) | 11% (9%, 13%) |
| TTIQ (Q=7 days) | 60-day Infections | 7% (6%, 7%) | 29% (27%, 32%) | 14% (12%, 16%) | 18% (16%, 20%) | 11% (9%, 12%) | 21% (19%, 23%) |
| TTIQ (Q=14 days) | 60-day Infections | 21% (19%, 22%) | 48% (45%, 51%) | 31% (28%, 34%) | 32% (29%, 35%) | 21% (19%, 23%) | 42% (39%, 45%) |
| No interventions | Total Infections | 48839 (48828, 48850) | 48839 (48826, 48852) | 48816 (48802, 48830) | 48863 (48852, 48874) | 48840 (48829, 48849) | 48838 (48827, 48850) |
| TI | Total Infections | 1% (0%, 1%) | 1% (0%, 1%) | 0% (0%, 1%) | 1% (1%, 1%) | 0% (0%, 0%) | 1% (1%, 1%) |
| TTI | Total Infections | 2% (2%, 2%) | 1% (1%, 2%) | 1% (1%, 1%) | 2% (2%, 2%) | 1% (1%, 1%) | 2% (2%, 2%) |
| TTIQ (Q=7 days) | Total Infections | 4% (4%, 5%) | 3% (3%, 3%) | 4% (3%, 4%) | 3% (3%, 4%) | 3% (3%, 3%) | 4% (4%, 4%) |
| TTIQ (Q=14 days) | Total Infections | 11% (10%, 12%) | 5% (4%, 5%) | 10% (9%, 10%) | 7% (6%, 7%) | 6% (6%, 7%) | 10% (9%, 11%) |

A.5.2 PHSM

The results for R0 of 7-12 are similar to a R0 of 2-7, but with smaller reductions in 60-day and total infections compared to the no interventions scenario. Due to the very high R0 values, the effectiveness of PHSM is very low as the disease spreads through the community rapidly.

Figure A7: Impact of public health and social measures compared to no interventions for different disease parameter ranges. Colour is used for illustrative purposes with light green meaning less infections averted, and dark green meaning more infections averted. (A) Impact on 60-day infections, (B) impact on total infections.

Table A8: Relative impact with uncertainty of different PHSM interventions compared to no interventions for 60-day and total infections.

| PHSM | Metric | Latent period (low) | Latent period (high) | Infectious duration (low) | Infectious duration (high) | Symptomatic fraction (low) | Symptomatic fraction (high) |
| --- | --- | --- | --- | --- | --- | --- | --- |
| No interventions | 60-day Infections | 48828 (48817, 48840) | 33440 (31768, 35031) | 41972 (40371, 43340) | 40394 (38837, 42061) | 41761 (40168, 43202) | 40681 (39051, 42393) |
| Masks | 60-day Infections | 0% (0%, 0%) | 14% (13%, 15%) | 5% (4%, 6%) | 6% (5%, 8%) | 5% (4%, 6%) | 6% (5%, 7%) |
| Work from home advised | 60-day Infections | 1% (1%, 1%) | 13% (12%, 14%) | 5% (4%, 6%) | 6% (5%, 8%) | 5% (4%, 6%) | 6% (5%, 7%) |
| Schools closed | 60-day Infections | 0% (0%, 0%) | 24% (21%, 26%) | 9% (7%, 11%) | 10% (8%, 12%) | 8% (7%, 10%) | 11% (9%, 13%) |
| Schools closed & work from home advised + masks | 60-day Infections | 5% (4%, 6%) | 64% (61%, 67%) | 27% (23%, 31%) | 30% (26%, 34%) | 26% (22%, 30%) | 31% (27%, 36%) |
| Lockdown + masks | 60-day Infections | 29% (26%, 33%) | 91% (90%, 92%) | 54% (49%, 59%) | 54% (48%, 59%) | 51% (46%, 56%) | 57% (52%, 61%) |
| No interventions | Total Infections | 48839 (48828, 48850) | 48839 (48826, 48852) | 48816 (48802, 48830) | 48863 (48852, 48874) | 48840 (48829, 48849) | 48838 (48827, 48850) |
| Masks | Total Infections | 0% (0%, 0%) | 0% (0%, 0%) | 0% (0%, 0%) | 0% (0%, 0%) | 0% (0%, 0%) | 0% (0%, 0%) |
| Work from home advised | Total Infections | 1% (1%, 1%) | 1% (1%, 1%) | 1% (1%, 1%) | 1% (1%, 1%) | 1% (1%, 1%) | 1% (1%, 1%) |
| Schools closed | Total Infections | 0% (0%, 0%) | 0% (0%, 0%) | 0% (0%, 0%) | 0% (0%, 0%) | 0% (0%, 0%) | 0% (0%, 0%) |
| Schools closed & work from home advised + masks | Total Infections | 2% (2%, 2%) | 2% (2%, 2%) | 2% (2%, 2%) | 1% (1%, 1%) | 2% (2%, 2%) | 2% (2%, 2%) |
| Lockdown + masks | Total Infections | 6% (5%, 6%) | 6% (6%, 6%) | 7% (6%, 7%) | 5% (5%, 5%) | 6% (5%, 6%) | 6% (6%, 6%) |

A.6 Pseudo-diseases

The analysis was repeated for four pseudo-diseases. The 5000 generated pathogens were filtered to fit into the parameter ranges that are similar to existing diseases (Table A9). The diseases were chosen to cover all possible values of R0 between the values of 1 and 18.

Table A9: Pathogen characteristics ranges that are similar to existing diseases (influenza, COVID-19, chickenpox, measles).

| Disease characteristic | Estimate | Source |
| --- | --- | --- |
| Influenza-like |  |  |
| R0 | 1-2 | Biggerstaff et al. [1] |
| Latent period | 1-6 days | US Hospital information [2] |
| Infectious duration | 1-10 days | US Hospital information [2] |
| Symptomatic fraction | 50-90% | Leung et al. [3] |
| COVID-19-like |  |  |
| R0 | 2-7 | Liu et al. [4] |
| Latent period | 2-8 days | Li et al. [5] |
| Infectious duration | 7-14 days | Qian et al. [6] |
| Symptomatic fraction | 50-75% | Ma et al. [7] |
| Chickenpox-like |  |  |
| R0 | 7-12 | Anderson & May [8] |
| Latent period | 8-14 days | US Centre for Disease Control [9] |
| Infectious duration | 4-8 days | National Centre for Infectious Diseases [10] |
| Symptomatic fraction | 80-100% | Gillard et al. [11] |
| Measles-like |  |  |
| R0 | 12-18 | Anderson & May [8] |
| Latent period | 8-14 days | US Centre for Disease Control [12] |
| Infectious duration | 6-14 days | US Centre for Disease Control [12] |
| Symptomatic fraction | 80-100% | Sonoda et al. [13] |

A.6.1 TTIQ

Figure A8 and Table A10 show the relative reduction in 60-day and total infections for different TTIQ scenarios given the four different pseudo-diseases. TTIQ interventions are most effective for influenza-like pathogens, likely due to the low R_0_ and relatively high symptomatic fraction. Furthermore, TTIQ interventions are more effective for chickenpox-like diseases, regardless of the higher R_0_ than COVID-19-like diseases due to the much higher fraction of symptomatic cases. TTIQ interventions have similar effectiveness for COVID-19-like and measles-like diseases due to two competing features: while COVID-19-like is easier to control due to the lower R_0_, measles-like are easier to detect given the higher symptomatic fraction.

Figure A8: Relative impact of different test, trace, isolation and quarantine (TTIQ) interventions compared to no interventions for different parameters combinations corresponding to an existing disease. Colour is used for illustrative purposes with light green meaning less infections averted, and dark green meaning more infections averted. (A) Impact on 60-day infections, (B) impact on total infections.

Table A10: Relative impact with uncertainty of different TTIQ interventions compared to no interventions for 60-day and total infections.

| TTIQ_label | Metric | Influenza-like | COVID-19-like | Chickenpox-like | Measles-like |
| --- | --- | --- | --- | --- | --- |
| No interventions | 60-day Infections | 13653 (9643, 18728) | 39112 (36790, 41525) | 32387 (29697, 34961) | 37940 (34268, 41388) |
| TI | 60-day Infections | 35% (22%, 51%) | 9% (7%, 12%) | 18% (16%, 21%) | 16% (12%, 21%) |
| TTI | 60-day Infections | 41% (27%, 58%) | 22% (18%, 25%) | 21% (18%, 23%) | 19% (15%, 24%) |
| TTIQ (Q=7 days) | 60-day Infections | 89% (80%, 96%) | 44% (40%, 48%) | 40% (37%, 43%) | 34% (29%, 39%) |
| TTIQ (Q=14 days) | 60-day Infections | 91% (84%, 97%) | 62% (58%, 66%) | 76% (74%, 78%) | 62% (57%, 66%) |
| No interventions | Total Infections | 26135 (21961, 29998) | 46047 (45435, 46611) | 48495 (48438, 48558) | 48855 (48836, 48873) |
| TI | Total Infections | 29% (18%, 40%) | 4% (3%, 4%) | 2% (2%, 2%) | 1% (1%, 1%) |
| TTI | Total Infections | 40% (26%, 55%) | 12% (11%, 14%) | 2% (2%, 3%) | 2% (2%, 2%) |
| TTIQ (Q=7 days) | Total Infections | 86% (78%, 93%) | 19% (17%, 21%) | 6% (6%, 7%) | 4% (3%, 4%) |
| TTIQ (Q=14 days) | Total Infections | 89% (81%, 96%) | 30% (28%, 33%) | 19% (18%, 21%) | 7% (6%, 9%) |

A.6.2 PHSM

Figure A9 and Table A11 show the relative reduction in 60-day and total infections for different PHSM scenarios given the four different pseudo-diseases. As seen sections A.2, A.3, A.4 and A.5, as R0 increases the effectiveness of PHSM decreases. There is a greater reduction in 60-day infections for chickenpox-like diseases compared to COVID-19-like diseases, as chickenpox-like have much larger latent periods. However, even though measles-like diseases also have a large latent period, due to the much higher R0 than COVID-19-like diseases the effectiveness of PHSM are not as great.

Figure A9: Impact of public health and social measures compared to no interventions for different parameters combinations corresponding to an existing disease. Colour is used for illustrative purposes with light green meaning less infections averted, and dark green meaning more infections averted. (A) Impact on 60-day infections, (B) impact on total infections.

Table A11: Relative impact with uncertainty of different PHSM interventions compared to no interventions for 60-day and total infections.

| PHSM | Metric | Influenza-like | COVID-19-like | Chickenpox-like | Measles-like |
| --- | --- | --- | --- | --- | --- |
| No interventions | 60-day Infections | 13653 (9643, 18728) | 39112 (36790, 41525) | 32387 (29697, 34961) | 37940 (34268, 41388) |
| Masks | 60-day Infections | 52% (38%, 66%) | 11% (9%, 14%) | 15% (13%, 17%) | 9% (7%, 12%) |
| Work from home advised | 60-day Infections | 48% (42%, 56%) | 15% (13%, 18%) | 15% (13%, 17%) | 9% (7%, 12%) |
| Schools closed | 60-day Infections | 62% (47%, 79%) | 16% (13%, 20%) | 22% (19%, 25%) | 16% (13%, 21%) |
| Schools closed & work from home advised + masks | 60-day Infections | 99% (99%, 99%) | 64% (58%, 71%) | 67% (63%, 71%) | 52% (45%, 60%) |
| Lockdown + masks | 60-day Infections | 100% (99%, 100%) | 90% (86%, 94%) | 93% (92%, 94%) | 86% (83%, 90%) |
| No interventions | Total Infections | 26135 (21961, 29998) | 46047 (45435, 46611) | 48495 (48438, 48558) | 48855 (48836, 48873) |
| Masks | Total Infections | 28% (22%, 36%) | 2% (2%, 3%) | 1% (1%, 1%) | 0% (0%, 0%) |
| Work from home advised | Total Infections | 41% (37%, 43%) | 7% (6%, 9%) | 2% (1%, 2%) | 1% (1%, 1%) |
| Schools closed | Total Infections | 37% (29%, 46%) | 3% (2%, 3%) | 0% (0%, 0%) | 0% (0%, 0%) |
| Schools closed & work from home advised + masks | Total Infections | 100% (99%, 100%) | 26% (21%, 32%) | 4% (4%, 5%) | 2% (1%, 2%) |
| Lockdown + masks | Total Infections | 100% (100%, 100%) | 58% (53%, 64%) | 16% (15%, 18%) | 6% (5%, 6%) |

A.7 Presymptomatic transmission

Presymptomatic transmission can be described as asymptomatic transmission by an individual who subsequently develops symptoms. While presymptomatic transmission was not explicitly modelled, from the parameters that have been sampled we attempted to approximate a pathogen with presymptomatic transmission as one that has a shorter latency period, a longer infectious period and a lower chance of an infection showing symptoms. We then compare the impact of TTIQ interventions for this and other pathogens with specific characteristics combinations, including: (1) Infectious soon after infection, low chance of symptoms, (2) Infectious soon after infection, high chance of symptoms, (3) Infectious delayed after infection, low chance of symptoms, (4) Infectious delayed after infection, high chance of symptoms.

Figure A10 and Table A12 show the relative reduction in 60-day and total infections for different TTIQ scenarios given the disease parameter combinations listed above. TTIQ is less effective for pathogens chosen to approximate presymptomatic transmission compared to pathogens with similar latent and infectious periods but with higher symptomatic fractions.

Figure A10: Relative impact of different test, trace, isolation and quarantine (TTIQ) interventions compared to no interventions. Each column represents different parameters combinations to demonstrate the impact of presymptomatic transmission on TTIQ measures. Colour is used for illustrative purposes with light green meaning less infections averted, and dark green meaning more infections averted. (A) Impact on 60-day infections, (B) impact on total infections.

Table A12: Relative impact with uncertainty of different TTIQ interventions compared to no interventions for 60-day and total infections.

| TTIQ | Metric | Latent (low), Infectious (high), Symptomatic (low) | Latent (high), Infectious (low), Symptomatic (high) | Latent (low), Infectious (high), Symptomatic (high) | Latent (high), Infectious (low), Symptomatic (low) |
| --- | --- | --- | --- | --- | --- |
| No interventions | 60-day Infections | 40127 (37919, 42006) | 17669 (16054, 19166) | 39550 (37487, 41458) | 17944 (16288, 19719) |
| TI | 60-day Infections | 6% (-3%, 13%) | 17% (5%, 30%) | 23% (16%, 29%) | 4% (-8%, 17%) |
| TTI | 60-day Infections | 17% (9%, 24%) | 19% (7%, 31%) | 35% (28%, 41%) | 6% (-6%, 19%) |
| TTIQ (Q=7 days) | 60-day Infections | 37% (30%, 43%) | 62% (56%, 68%) | 60% (55%, 65%) | 28% (18%, 38%) |
| TTIQ (Q=14 days) | 60-day Infections | 52% (47%, 58%) | 80% (76%, 83%) | 82% (79%, 85%) | 39% (30%, 48%) |
| No interventions | Total Infections | 46011 (45580, 46424) | 44872 (44445, 45232) | 45494 (44924, 46023) | 45074 (44683, 45424) |
| TI | Total Infections | 2% (1%, 4%) | 5% (3%, 6%) | 13% (10%, 15%) | 1% (0%, 2%) |
| TTI | Total Infections | 11% (9%, 13%) | 6% (5%, 8%) | 24% (21%, 28%) | 2% (1%, 4%) |
| TTIQ (Q=7 days) | Total Infections | 17% (15%, 19%) | 20% (18%, 22%) | 31% (28%, 35%) | 9% (7%, 10%) |
| TTIQ (Q=14 days) | Total Infections | 25% (23%, 28%) | 43% (41%, 46%) | 59% (55%, 63%) | 16% (14%, 18%) |

**References**

1.	Biggerstaff, M., et al., *Estimates of the reproduction number for seasonal, pandemic, and zoonotic influenza: a systematic review of the literature.* BMC Infect Dis, 2014. **14**: p. 480.

2.	MedPark Hospital. *Influenza A: Causes, Symptoms, Treatments, Recovery*. 2026 [cited 2026 13 May].

3.	Leung, N.H., et al., *The fraction of influenza virus infections that are asymptomatic: a systematic review and meta-analysis.* Epidemiology, 2015. **26**(6): p. 862-872.

4.	Liu, Y. and J. Rocklov, *The reproductive number of the Delta variant of SARS-CoV-2 is far higher compared to the ancestral SARS-CoV-2 virus.* J Travel Med, 2021. **28**(7).

5.	Li, B., et al., *Viral infection and transmission in a large, well-traced outbreak caused by the SARS-CoV-2 Delta variant.* Nat Commun, 2022. **13**(1): p. 460.

6.	Qian, G.Q., et al., *Duration of SARS-CoV-2 viral shedding during COVID-19 infection.* Infect Dis (Lond), 2020. **52**(7): p. 511-512.

7.	Ma, Q., et al., *Global percentage of asymptomatic SARS-CoV-2 infections among the tested population and individuals with confirmed COVID-19 diagnosis: a systematic review and meta-analysis.* JAMA network open, 2021. **4**(12): p. e2137257.

8.	Anderson, R.M. and R.M. May, *Infectious diseases of humans: dynamics and control*. 1991: Oxford university press.

9.	US Centre for Disease Control. *Clinical Overview of Chickenpox (Varicella)*. 2024 [cited 2026 13 May].

10.	National Centre for Infectious Diseases. *Chickenpox*. 2026 [cited 2026 13 May].

11.	Gillard, P., M. Povey, and S. Carryn, *Clinically- versus serologically-identified varicella: A hidden infection burden. A ten-year follow-up from a randomized study in varicella-endemic countries.* Hum Vaccin Immunother, 2021. **17**(10): p. 3747-3756.

12.	US Centre for Disease Control. *Measles Clinical Diagnosis Fact Sheet*. 2025 [cited 2026 13 May].

13.	Sonoda, S. and T. Nakayama, *Detection of measles virus genome in lymphocytes from asymptomatic healthy children.* J Med Virol, 2001. **65**(2): p. 381-7.
